# Supplementary material for: Diagnostic performance of PfHRP2/pLDH malaria rapid diagnostic tests in elimination setting, northwest Ethiopia
Source: PLOS Glob Public Health. 2023 Jul 10;3(7):e0001879. doi: 10.1371/journal.pgph.0001879 (PMC10332618; doi:10.1371/journal.pgph.0001879)
Supplement: Attachment — Submitted filename: Response to reviewers.docx [file pgph.0001879.s002.docx]

**Authors’ Response to Academic Editor’s and Reviewers’ Comments:**

**Manuscript No:** PGPH-D-22-01932

**Title:** Diagnostic performance of PfHRP2/pLDH malaria rapid diagnostic tests in elimination setting, northwest Ethiopia.

We would like to thank the academic editor and the reviewers for the thorough review of our manuscript, encouraging words, helpful comments, and the opportunity to resubmit a revised copy of the manuscript. We have addressed all the comments as suggested by the academic editor and reviewers in our manuscript, as indicated below:

**Journal Requirements:**

1. Please indicate the full and correct funding information for your study and confirm the order in which funding contributions should appear.

**Authors’ Response**:

The funding information for this study is indicated and listed according to their contribution in the revised manuscript. Now, the journal requirements have been fulfilled.

**Reviewer's Responses to Questions**
1. Does this manuscript meet PLOS Global Public Health’s [publication criteria](https://journals.plos.org/globalpublichealth/s/criteria-for-publication)? Is the manuscript technically sound, and do the data support the conclusions? The manuscript must describe methodologically and ethically rigorous research with conclusions that are appropriately drawn based on the data presented.

Reviewer #1: Yes

Reviewer #2: Partly

2. Has the statistical analysis been performed appropriately and rigorously?

Reviewer #1: No

Reviewer #2: Yes

3. Have the authors made all data underlying the findings in their manuscript fully available (please refer to the Data Availability Statement at the start of the manuscript PDF file)?
The [PLOS Data policy](https://journals.plos.org/globalpublichealth/s/data-availability) requires authors to make all data underlying the findings described in their manuscript fully available without restriction, with rare exception. The data should be provided as part of the manuscript or its supporting information, or deposited to a public repository. For example, in addition to summary statistics, the data points behind means, medians and variance measures should be available. If there are restrictions on publicly sharing data—e.g. participant privacy or use of data from a third party—those must be specified.

Reviewer #1: Yes

Reviewer #2: No

4. Is the manuscript presented in an intelligible fashion and written in standard English?
PLOS Global Public Health does not copyedit accepted manuscripts, so the language in submitted articles must be clear, correct, and unambiguous. Any typographical or grammatical errors should be corrected at revision, so please note any specific errors here.

Reviewer #1: Yes

Reviewer #2: Yes

**5. Review Comments to the Author**
Please use the space provided to explain your answers to the questions above. You may also include additional comments for the author, including concerns about dual publication, research ethics, or publication ethics. (Please upload your review as an attachment if it exceeds 20,000 characters)

Reviewer #1: Thank you for the opportunity to review this paper. Here are some comments for authors' consideration, hopefully to further strengthen the paper.

**Major comments:**
1. CareStart is a trademark protected by applicable laws. Please strongly justify the use of trademarked names, instead of just saying PfHRP2/pLDH, in your manuscript, otherwise please omit them.

**Authors’ Response**:

We agreed with the comment, and now the tradename “CareStart” has been removed from the title of the manuscript.

2. Please elaborate what is known about malaria transmission intensity, parasite and vector diversity in the catchment area and population served by Andassa Health Center (AHC). This critical for a better understanding of the diagnostic performance of PfHRP2/pLDH.

**Authors’ Response**:

We elaborated the malaria transmission intensity, parasite, and vector in the study area on page No.5 from lines 95 to 105 in the manuscript.

3. A lot more descriptive data needs to be shown before the diagnostic performance results can be credibly interpreted. Please add a table first to describe your study population ( children vs. adults) and study site ( non-malaria areas vs malarious areas) and stratify the diagnostic performance findings across these variables.

**Authors’ Response**:

Descriptive data added in the revised manuscript.

4. The authors state AHC was purposively selected, so please justify the use of inferential statistics in your analysis? If your sample was purposively selected the 95% confidence interval and p-values become uninterpretable. Please justify or revise accordingly.

**Authors’ Response:**

Among the available health centers in the study areas, Andassa health center was selected because of the feasibility in terms of the data collection, sample storage, handling, and transportation to the regional reference laboratory and this health center is adjacent to the parasite clearance intervention village. There is no health center in the intervention village.

**Minor comments:**
1. The term “rate” should not be used to describe proportions or percentages. In descriptive epidemiology the term “rate” implies person-time, which is not the case in this manuscript. Please appropriately revise.

**Authors’ Response:**

We used the term “rate” to explain false-negative and false-positive rates, and test positivity rate which are common ways of expression in diagnostic studies cited. Yes, we agreed that “rate” in epidemiology expresses person-time like incidence, prevalence, etc.
2. Please define the term ‘elimination settings”.

**Authors’ Response:**

There is no standard definition for “Elimination settings”. Elimination setting/s is/are geographic areas targeted for the malaria elimination program.
3. Line 99: what does “als” mean. One should not have to guess what it means.

**Authors’ Response**:

In the revised manuscript “asl” above sea level is written in full words.
4. Please support suspected malaria case definition with relevant references.

**Authors’ Response**:

A suspected malaria case is well defined in the “participants and eligibility” section of the manuscript on pages 5 and 6 lines 113-118 with its reference.
5. Please clearly state how consent was sought from under-age population (children).

**Authors’ Response**:

Written assent was obtained in the age group between 12-17 in addition to consent from their parents/guardians. The ethical considerations were revised to clearly state how consent was obtained in children.
6. Line 126: the test does not detect plasmodium it detects certain antigens of the plasmodium. Please rephrase for clarity and accuracy.

**Authors’ Response**:

The sentence was rephrased accordingly.

7. Laboratory methods: Please support all your laboratory methods (RDTs, microscopy and PCR) with references to reflect that standardized protocols were followed. Any deviations from standardized protocols should be clearly stated.

**Authors’ Response:**

All the laboratory procedures strictly followed the standard operating procedures (SOPs) and instructions of the manufacturer. This is clearly stated in the method part from pages 6 to 8. No deviations were reported.
8. Formulas for diagnostic accuracy should be supported with relevant references.

**Authors’ Response**: Reference cited in the formula of diagnostic accuracy
9. Fig1 and methods section should also clearly state and show how many suspected malaria cases were screened and how many were excluded for receiving antimalarials within 4 weeks before arriving at the sample size of 310.

**Authors’ Response:**

A total of 310 febrile patients who had fulfilled the inclusion criteria were screened and enrolled in the study. Those febrile patients who didn’t fulfill the criteria were excluded and not counted/recorded in the study.
10. Table 4. Please clearly justify class interval used in the frequency table. Parasite density cutoffs and class intervals needs to reflect underlying biological, pathological, or operational basis.

**Authors’ Response:**

The interval we used were taken from previous studies and cited in the manuscript.

Reviewer #2: 1. In discussing study area, line 97 describes Andassa health center as a representative of all transmission settings. Please clarify this.

**Authors’ Response:**

Now the study area is well described in the revised manuscript by considering the comments provided.

2. Please include a picture of the nomogram as a figure in the publication

**Authors’ Response:**

The nomograph is not our original work. We cited the original reference if anyone interested to see the picture.
3. In the methods discussing parasite density calculation, lines 169-174 does not include information on how the standard curve generated via PCR was used to arrive at contents of table 4

**Authors’ Response:**

The protocol for parasite density calculation for *Plasmodium falciparum* is well described on page 8 from lines 173 to 178.
4. Table 4 should be part of results and not discussion.

**Authors’ Response:**

We agreed and Table 4 is moved to the result section.
5. Specific mention should be made about the benefits and opportunities for use of Malaria RDTs in your discussions and conclusion to avoid giving the impression that they are diagnostically useless

**Authors’ Response:**

**We concluded that:** The diagnostic performance of *Pf*HRP2/pLDH CareStart RDTs in detecting malaria parasites among febrile patients in the study area was below the recommended WHO standard. The limited diagnostic performance of RDTs in the malaria elimination area undoubtedly affects the impact of malaria parasite clearance interventions. Therefore, parasite clearance intervention like targeted mass drug administration with antimalarial drugs is recommended to back up the limited diagnostic performance of the RDT or replace the existing malaria RDTs with more sensitive, field-deployable, and affordable diagnostic tests.

6. PLOS authors have the option to publish the peer review history of their article ([what does this mean?](https://journals.plos.org/globalpublichealth/s/editorial-and-peer-review-process#loc-peer-review-history)). If published, this will include your full peer review and any attached files.


**Do you want your identity to be public for this peer review?** If you choose “no”, your identity will remain anonymous but your review may still be made public.


For information about this choice, including consent withdrawal, please see our [Privacy Policy](https://www.plos.org/privacy-policy).

Reviewer #1: No

Reviewer #2: **Yes:** Anthony Agbakizu Ahumibe
