# Supplementary material for: Diagnostic performance of PfHRP2/pLDH malaria rapid diagnostic tests in elimination setting, northwest Ethiopia
Source: PLOS Glob Public Health. 2023 Jul 10;3(7):e0001879. doi: 10.1371/journal.pgph.0001879 (PMC10332618; doi:10.1371/journal.pgph.0001879)
Supplement: Attachment — Submitted filename: Response to reviewers.docx [file pgph.0001879.s003.docx]

**Authors’ Response to Academic Editor’s and Reviewers’ Comments:**

**Manuscript No: PGPH-D-22-01932R1**

**Title: Diagnostic performance of PfHRP2/pLDH malaria rapid diagnostic tests in elimination setting, northwest Ethiopia.**

We would like to thank the academic editor and the reviewers for the thorough review of our revised manuscript, and the opportunity to resubmit a revised manuscript. In our previous revised manuscript with track change, we failed to cite two articles but not in the unmarked version of the revised manuscript. Now, we have corrected this, and the reference lists are corrected and completed. We have addressed all the comments as suggested by the academic editor as indicated below:

**Journal Requirements:**
Please review your reference list to ensure that it is complete and correct. If you have cited papers that have been retracted, please include the rationale for doing so in the manuscript text, or remove these references and replace them with relevant current references. Any changes to the reference list should be mentioned in the rebuttal letter that accompanies your revised manuscript. If you need to cite a retracted article, indicate the article’s retracted status in the References list and also include a citation and full reference for the retraction notice.

**Authors’ Response:**

We have checked all the lists of references and now the references are completed and corrected. Two articles (18 and 19) were not inserted in the previously revised manuscript with track change, now these articles have been inserted and completed. To our knowledge, we haven’t used retracted articles/papers. We have checked that all cited articles/papers are not retracted. We are happy to remove the idea/s and reference/s of any retracted article/s if you detect it.

**Additional Editor Comments (if provided):**

***Please revise the article as this revised version of draft has errors. For examples- Line 199: references is still missing, table 4 is still in discussion section etc.


**Authors’ Response:**

We have inserted a reference at line199 and table 4 has been moved and inserted in the result section.

Reviewers' comments:


[NOTE: If reviewer comments were submitted as an attachment file, they will be attached to this email and accessible via the submission site. Please log into your account, locate the manuscript record, and check for the action link "View Attachments". If this link does not appear, there are no attachment files.]
